# *cis*-Di-μ-oxido-bis­[(*N*,*N*-diethyl­dithio­carbamato-κ^2^ *S*,*S*′)oxidomolybdenum(V)](*Mo*—*Mo*) tetra­hydro­furan monosolvate

**DOI:** 10.1107/S1600536811003187

**Published:** 2011-01-29

**Authors:** José A. Fernandes, Filipe A. Almeida Paz, Carlos C. Romão

**Affiliations:** a Department of Chemistry, University of Aveiro, CICECO, 3810-193 Aveiro, Portugal; b Instituto de Tecnologia Química e Biológica, Universidade Nova de Lisboa, Av. da República, EAN, 2780-157 Oeiras, Portugal

## Abstract

The title compound, [Mo_2_(C_5_H_10_NS_2_)_2_O_4_]·C_4_H_8_O, can be readily prepared in tetra­hydro­furan (THF) by an oxidation reaction between the Mo^IV^ precursor [MoO(S_2_CNEt_2_)_2_] with [ReMeO_3_]. The compound is an axially symmetric Mo^V^ dimer (2 symmetry), in which the metal atoms exhibit a distorted square-pyramidal coordination environment. A THF mol­ecule was found to be equally disordered over two symmetry-related sites (around a twofold rotation axis), *trans*-coordinated to the apical oxido group and weakly inter­acting with the Mo^V^ atoms [Mo—O = 2.6213 (19) Å]. In the crystal, some weak C—H⋯O inter­actions occur between the terminal oxido and neighbouring —CH_3_ groups of an adjacent [Mo(μ-O)O(S_2_CNEt_2_)]_2_ unit.

## Related literature

For applications of dithio­carbamate compounds, see: Tiekink (2008 ▶); Zhao *et al.* (2005 ▶). For the synthesis of the Mo^IV^ precursor, [MoO(S_2_CNEt_2_)_2_], see: Jowitt & Mitchell (1969 ▶). For the synthesis of unsolvated [Mo(μ-O)O(S_2_CNEt_2_)]_2_, see: Ricard *et al.* (1975 ▶). For previous reports on dithio­carbamate compounds from our research groups, see: Drew *et al.* (1998 ▶); Romão & Royo (2002 ▶); Almeida Paz *et al.* (2003 ▶). For molybdenum dimers with long Mo—O_THF_ bonds, see: Cotton *et al.* (1978 ▶, 1992 ▶); Cotton & Su (1995 ▶). For a description of the Cambridge Structural Database, see: Allen (2002 ▶).
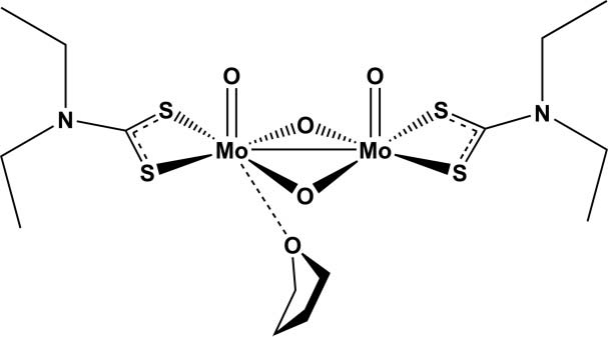


## Experimental

### 

#### Crystal data

[Mo_2_(C_5_H_10_NS_2_)_2_O_4_]·C_4_H_8_O
*M* *_r_* = 624.50
Monoclinic, 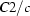

*a* = 12.8695 (7) Å
*b* = 12.6025 (7) Å
*c* = 14.4579 (8) Å
β = 94.184 (3)°
*V* = 2338.6 (2) Å^3^
*Z* = 4
Mo *K*α radiation
μ = 1.46 mm^−1^
*T* = 150 K
0.12 × 0.12 × 0.08 mm

#### Data collection

Bruker X8 KappaCCD APEXII diffractometer
Absorption correction: multi-scan (*SADABS*; Sheldrick, 1998 ▶) *T*_min_ = 0.845, *T*_max_ = 0.893
48139 measured reflections
5649 independent reflections
4661 reflections with *I* > 2σ(*I*)
*R*_int_ = 0.033

#### Refinement

*R*[*F* ^2^ > 2σ(*F* ^2^)] = 0.024
*wR*(*F* ^2^) = 0.054
*S* = 1.03
5649 reflections
149 parameters
5 restraints
H-atom parameters constrained
Δρ_max_ = 0.74 e Å^−3^
Δρ_min_ = −0.96 e Å^−3^

### 

Data collection: *APEX2* (Bruker, 2006 ▶); cell refinement: *SAINT-Plus* (Bruker, 2005 ▶); data reduction: *SAINT-Plus*; program(s) used to solve structure: *SHELXTL* (Sheldrick, 2008 ▶); program(s) used to refine structure: *SHELXTL*; molecular graphics: *DIAMOND* (Brandenburg, 2009 ▶); software used to prepare material for publication: *SHELXTL*.

## Supplementary Material

Crystal structure: contains datablocks global, I. DOI: 10.1107/S1600536811003187/pk2294sup1.cif

Structure factors: contains datablocks I. DOI: 10.1107/S1600536811003187/pk2294Isup2.hkl

Additional supplementary materials:  crystallographic information; 3D view; checkCIF report

## Figures and Tables

**Table 1:** Selected bond lengths (Å)

Mo1—Mo1^i^	2.5591 (2)
Mo1—S1	2.4788 (4)
Mo1—S2	2.4680 (4)
Mo1—O1	1.9586 (9)
Mo1—O1^i^	1.9472 (9)
Mo1—O2	1.6826 (10)

Symmetry code: (i) 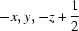.

**Table 2:** Hydrogen-bond geometry (Å, °)

*D*—H⋯*A*	*D*—H	H⋯*A*	*D*⋯*A*	*D*—H⋯*A*
C2—H2*A*⋯O2^ii^	0.99	2.57	3.2687 (17)	127
C4—H4*B*⋯O2^iii^	0.99	2.49	3.2443 (17)	133

Symmetry codes: (ii) 
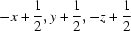
; (iii) 
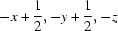
.

**Fig. 1.**
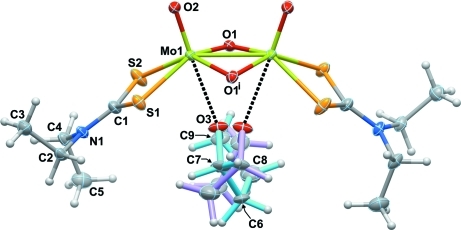
Molecular unit of the title compound showing all non-hydrogen atoms as thermal ellipsoids drawn at the 40% probability level and hydrogen atoms as small spheres with arbitrary radii. Atoms composing the asymmetric unit are highlighted by showing their respective label. The two possible locations for the disordered THF molecule are depicted with different colours for the bonds. For selected bond lengths see Table 1. Symmetry transformation used to generate equivalent atoms: (i) -x, y, 0.5 - z.

**Fig. 2.**
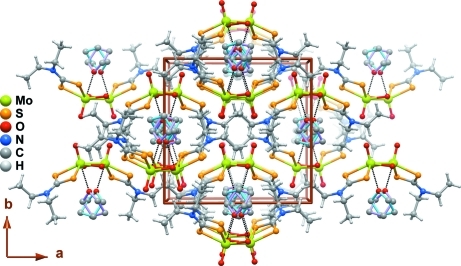
Crystal packing of the title compound viewed in perspective along the [001] direction of the unit cell. The two possible locations for the disordered THF molecule are depicted with different colours for the bonds (hydrogen atoms associated with these moieties have been omitted for clarity).

## supplementary crystallographic information

### Comment

Dithiocarbamates and their metal complexes have several applications in the treatment of some diseases (*e.g.*, Wilson's disease and alcoholism) and industry processes (*e.g.*, vulcanization of rubber and pesticides) (Tiekink, 2008). These molecules have already been used in the functionalization of gold nanoparticles (Zhao *et al.*, 2005). Following our interest in the preparation of dithiocarbamate metal complexes (Drew *et al.*, 1998; Romão *et al.*, 2002; Almeida Paz *et al.*, 2003), we attempted the preparation of a heterobimetallic compound from the reaction of [MoO(S_2_CNEt_2_)_2_] and [ReMeO_3_]. The sole product was an oxidation product whose structure we wish to report here: [Mo(µ-O)O(S_2_CNEt_2_)]_2_^.^THF. We note that the corresponding unsolvated compound, [Mo(µ-O)O(S_2_CNEt_2_)]_2_, was previously reported by Ricard *et al.* (1975).

The title compound (see Scheme) is an axially symmetrical dimer of molybdenum(V) formed by way of two µ-oxido bridges, and with a short distance interaction with a tetrahydrofuran (THF) molecule, which was found to be disordered over two sites (symmetry-related by a twofold rotation axis). The asymmetric unit comprises one half of the molybdenum(V) dimer, and a half-occupied THF molecule (Figure 1). The coordination geometry around the metal centre can be envisaged as a highly distorted square pyramid (Table 1) with a terminal oxido ligand at the apex while the basal plane is occupied by two symmetry-equivalent µ-oxido bridges and a chelating dithiocarbamato ligand {MoO_3_S_2_}. The dimer also has a Mo—Mo direct bond with an intermetallic distance of 2.5591 (2) Å. A weakly-bonded THF molecule is *trans* to the apical oxido group.

The disordered THF molecule interacts weakly with the metal centre with the measured Mo···O distance being considerably longer [2.6213 (19) Å] than those typically found in related structures. Nevertheless, from a survey in the Cambridge Structural Database (Allen, 2002) we found 4 structures which have longer Mo—O_THF_ bonds than those of the title compound. These compounds correspond to isostructural dimers, each with four bridging carboxylates or dithiocarboxylates, very short Mo—Mo distances, and with THF acting as an axial ligand (Cotton *et al.*, 1978, 1992, 1995).

Individual [Mo(µ-O)O(S_2_CNEt_2_)]_2_^.^THF complexes close pack in the solid state driven by the need to effectively fill the available space (Figure 2). Some weak C—H···O interactions are present connecting the terminal oxido and neighbouring —CH_3_ groups of an adjacent [Mo(µ-O)O(S_2_CNEt_2_)]_2_ entity (not shown; see Table 2 for geometrical details).

### Experimental

The precursor [MoO(S_2_CNEt_2_)_2_] (1) was prepared using a published method (Jowitt & Mitchell, 1969). [ReMeO_3_] (MTO) was purchased from Sigma-Aldrich (71–76% of Re content), and used without any further purification. All manipulations were carried out by using standard Schlenk line and drybox techniques in an atmosphere of N_2_. THF was distilled from Na/benzophenone.

A solution of MTO (0.18 g, 0.73 mmol) in THF (10 ml) was added to a solution of 1 (0.30 g, 0.73 mmol) in THF (15 ml). After stirring magnetically for 5 h at ambient temperature, a green solution and a green solid were obtained. The volume of the mixture was reduced to half by vacuum evaporation and further precipitation was forced by cooling to -30 °C. The solid product was dissolved in the minimum amount of hot THF and submitted to slow cooling to -30 °C. Yellow crystals of the title compound suitable for single-crystal X-ray diffraction were directly isolated and preserved in N_2_ atmosphere prior to data collection.

### Refinement

Hydrogen atoms bound to carbon were placed in calculated positions and were included in the final structural model in riding-motion approximation with C—H = 0.99 (for the —CH_2_ moieties) or 0.98 Å (for the terminal —CH_3_ groups). The isotropic thermal displacement parameters for these atoms were fixed at 1.2 or 1.5×*U*_eq_ of the respective parent carbon atom (for —CH_2_— and —CH_3_, respectively).

The C—C and C—O bonds of the disordered THF molecule (modeled with a fixed 50% rate of occupancy for each location) were restrained to common refineable distances in order to ensure a chemically reasonable geometry for this moiety.

### Crystal data

**Table:**

[Mo_2_(C_5_H_10_NS_2_)_2_O_4_]·C_4_H_8_O	*F*(000) = 1256
*M**_r_* = 624.50	*D*_x_ = 1.774 Mg m^−^^3^
Monoclinic, *C*2/*c*	Mo *K*α radiation, λ = 0.71073 Å
Hall symbol: -C 2yc	Cell parameters from 9063 reflections
*a* = 12.8695 (7) Å	θ = 2.6–35.3°
*b* = 12.6025 (7) Å	µ = 1.46 mm^−^^1^
*c* = 14.4579 (8) Å	*T* = 150 K
β = 94.184 (3)°	Prism, yellow
*V* = 2338.6 (2) Å^3^	0.12 × 0.12 × 0.08 mm
*Z* = 4	

### Data collection

**Table:**

Bruker X8 KappaCCD APEXII diffractometer	5649 independent reflections
Radiation source: fine-focus sealed tube	4661 reflections with *I* > 2σ(*I*)
graphite	*R*_int_ = 0.033
ω and φ scans	θ_max_ = 36.3°, θ_min_ = 3.5°
Absorption correction: multi-scan (*SADABS*; Sheldrick, 1998)	*h* = −21→21
*T*_min_ = 0.845, *T*_max_ = 0.893	*k* = −20→20
48139 measured reflections	*l* = −23→24

### Refinement

**Table:**

Refinement on *F*^2^	Primary atom site location: structure-invariant direct methods
Least-squares matrix: full	Secondary atom site location: difference Fourier map
*R*[*F*^2^ > 2σ(*F*^2^)] = 0.024	Hydrogen site location: inferred from neighbouring sites
*wR*(*F*^2^) = 0.054	H-atom parameters constrained
*S* = 1.03	*w* = 1/[σ^2^(*F*_o_^2^) + (0.0219*P*)^2^ + 2.1445*P*] where *P* = (*F*_o_^2^ + 2*F*_c_^2^)/3
5649 reflections	(Δ/σ)_max_ = 0.001
149 parameters	Δρ_max_ = 0.74 e Å^−^^3^
5 restraints	Δρ_min_ = −0.96 e Å^−^^3^

### Special details

**Table:**

Geometry. All e.s.d.'s (except the e.s.d. in the dihedral angle between two l.s. planes) are estimated using the full covariance matrix. The cell e.s.d.'s are taken into account individually in the estimation of e.s.d.'s in distances, angles and torsion angles; correlations between e.s.d.'s in cell parameters are only used when they are defined by crystal symmetry. An approximate (isotropic) treatment of cell e.s.d.'s is used for estimating e.s.d.'s involving l.s. planes.
Refinement. Refinement of *F*^2^ against ALL reflections. The weighted *R*-factor *wR* and goodness of fit *S* are based on *F*^2^, conventional *R*-factors *R* are based on *F*, with *F* set to zero for negative *F*^2^. The threshold expression of *F*^2^ > σ(*F*^2^) is used only for calculating *R*-factors(gt) *etc*. and is not relevant to the choice of reflections for refinement. *R*-factors based on *F*^2^ are statistically about twice as large as those based on *F*, and *R*- factors based on ALL data will be even larger.

### Fractional atomic coordinates and isotropic or equivalent isotropic displacement parameters (Å^2^)

**Table:**

	*x*	*y*	*z*	*U*_iso_*/*U*_eq_	Occ. (<1)
Mo1	0.081183 (8)	0.231212 (9)	0.203909 (7)	0.01717 (3)	
S1	0.25495 (3)	0.31525 (3)	0.23038 (2)	0.02555 (7)	
S2	0.11812 (3)	0.33069 (4)	0.06297 (2)	0.03297 (9)	
O1	−0.06648 (7)	0.24910 (7)	0.16391 (6)	0.02023 (17)	
O2	0.11161 (8)	0.10400 (8)	0.18310 (7)	0.0273 (2)	
N1	0.30133 (9)	0.43361 (9)	0.08463 (7)	0.0222 (2)	
C1	0.23528 (10)	0.36816 (11)	0.12027 (9)	0.0224 (2)	
C2	0.40421 (10)	0.45692 (12)	0.13224 (9)	0.0250 (3)	
H2A	0.3998	0.4513	0.2001	0.030*	
H2B	0.4248	0.5304	0.1179	0.030*	
C3	0.48586 (12)	0.38032 (14)	0.10190 (12)	0.0336 (3)	
H3A	0.4671	0.3078	0.1187	0.050*	
H3B	0.5538	0.3986	0.1328	0.050*	
H3C	0.4895	0.3850	0.0345	0.050*	
C4	0.27763 (12)	0.48736 (12)	−0.00497 (9)	0.0288 (3)	
H4A	0.2232	0.4471	−0.0420	0.035*	
H4B	0.3409	0.4887	−0.0400	0.035*	
C5	0.24037 (16)	0.59952 (15)	0.00879 (12)	0.0423 (4)	
H5A	0.1750	0.5980	0.0396	0.063*	
H5B	0.2288	0.6345	−0.0516	0.063*	
H5C	0.2931	0.6387	0.0473	0.063*	
O3	0.02301 (17)	0.42667 (15)	0.23443 (16)	0.0298 (5)	0.50
C6	0.0064 (3)	0.5996 (3)	0.2983 (3)	0.0398 (8)	0.50
H6X	0.0481	0.6611	0.2799	0.048*	0.50
H6Y	−0.0215	0.6149	0.3589	0.048*	0.50
C7	0.0722 (3)	0.4986 (3)	0.3034 (3)	0.0370 (9)	0.50
H7X	0.1447	0.5143	0.2891	0.044*	0.50
H7Y	0.0732	0.4669	0.3661	0.044*	0.50
C8	−0.0831 (4)	0.5757 (3)	0.2237 (3)	0.0552 (11)	0.50
H8X	−0.1463	0.5508	0.2523	0.066*	0.50
H8Y	−0.1008	0.6391	0.1854	0.066*	0.50
C9	−0.0384 (3)	0.4905 (3)	0.1677 (3)	0.0346 (8)	0.50
H9X	−0.0943	0.4476	0.1353	0.042*	0.50
H9Y	0.0058	0.5209	0.1211	0.042*	0.50

### Atomic displacement parameters (Å^2^)

**Table:**

	*U*^11^	*U*^22^	*U*^33^	*U*^12^	*U*^13^	*U*^23^
Mo1	0.01784 (5)	0.01611 (5)	0.01787 (5)	−0.00165 (3)	0.00340 (3)	0.00016 (3)
S1	0.02101 (13)	0.03126 (18)	0.02375 (14)	−0.00642 (12)	−0.00270 (11)	0.00826 (12)
S2	0.02936 (16)	0.0438 (2)	0.02449 (15)	−0.01877 (15)	−0.00690 (12)	0.01249 (14)
O1	0.0200 (4)	0.0193 (4)	0.0216 (4)	−0.0037 (3)	0.0031 (3)	−0.0016 (3)
O2	0.0301 (5)	0.0201 (5)	0.0334 (5)	−0.0007 (4)	0.0140 (4)	−0.0024 (4)
N1	0.0239 (5)	0.0239 (6)	0.0188 (4)	−0.0081 (4)	0.0026 (4)	−0.0002 (4)
C1	0.0219 (5)	0.0235 (6)	0.0216 (5)	−0.0054 (4)	0.0010 (4)	0.0027 (4)
C2	0.0239 (6)	0.0270 (7)	0.0243 (6)	−0.0102 (5)	0.0024 (4)	−0.0024 (5)
C3	0.0262 (6)	0.0337 (8)	0.0412 (8)	−0.0055 (6)	0.0034 (6)	−0.0031 (6)
C4	0.0357 (7)	0.0315 (8)	0.0192 (5)	−0.0125 (6)	0.0027 (5)	0.0032 (5)
C5	0.0556 (11)	0.0344 (9)	0.0356 (8)	−0.0022 (8)	−0.0049 (7)	0.0071 (7)
O3	0.0374 (15)	0.0189 (9)	0.0305 (13)	0.0046 (7)	−0.0149 (9)	−0.0052 (8)
C6	0.0471 (19)	0.0201 (14)	0.054 (2)	−0.0070 (13)	0.0157 (16)	−0.0181 (13)
C7	0.044 (2)	0.0296 (19)	0.036 (2)	−0.0117 (16)	−0.0057 (15)	−0.0132 (16)
C8	0.060 (3)	0.033 (2)	0.074 (3)	0.0083 (18)	0.005 (2)	0.0059 (19)
C9	0.039 (2)	0.0247 (16)	0.038 (2)	0.0112 (15)	−0.0093 (15)	0.0032 (15)

### Geometric parameters (Å, °)

**Table:**

Mo1—Mo1^i^	2.5591 (2)	C4—C5	1.510 (3)
Mo1—S1	2.4788 (4)	C4—H4A	0.9900
Mo1—S2	2.4680 (4)	C4—H4B	0.9900
Mo1—O1	1.9586 (9)	C5—H5A	0.9800
Mo1—O1^i^	1.9472 (9)	C5—H5B	0.9800
Mo1—O2	1.6826 (10)	C5—H5C	0.9800
Mo1—O3	2.6213 (19)	O3—C9	1.446 (4)
S1—C1	1.7276 (13)	O3—C7	1.457 (4)
S2—C1	1.7322 (13)	O3—Mo1^i^	2.972 (2)
O1—Mo1^i^	1.9472 (9)	C6—C7	1.527 (5)
O2—Mo1^i^	3.4621 (10)	C6—C8	1.549 (5)
N1—C1	1.3161 (16)	C6—H6X	0.9900
N1—C4	1.4739 (17)	C6—H6Y	0.9900
N1—C2	1.4766 (17)	C7—H7X	0.9900
C2—C3	1.515 (2)	C7—H7Y	0.9900
C2—H2A	0.9900	C8—C9	1.485 (5)
C2—H2B	0.9900	C8—H8X	0.9900
C3—H3A	0.9800	C8—H8Y	0.9900
C3—H3B	0.9800	C9—H9X	0.9900
C3—H3C	0.9800	C9—H9Y	0.9900
			
O1^i^—Mo1—O1	96.59 (4)	C5—C4—H4A	109.4
O1—Mo1—O3	70.04 (6)	N1—C4—H4B	109.4
O1^i^—Mo1—O3	71.13 (6)	C5—C4—H4B	109.4
O1—Mo1—S1	147.29 (3)	H4A—C4—H4B	108.0
O1^i^—Mo1—S1	87.14 (3)	C4—C5—H5A	109.5
O1—Mo1—S2	86.50 (3)	C4—C5—H5B	109.5
O1^i^—Mo1—S2	142.17 (3)	H5A—C5—H5B	109.5
O2—Mo1—O1	106.88 (5)	C4—C5—H5C	109.5
O2—Mo1—O1^i^	109.05 (5)	H5A—C5—H5C	109.5
O2—Mo1—O3	176.86 (6)	H5B—C5—H5C	109.5
O2—Mo1—S1	102.45 (4)	C9—O3—C7	107.4 (2)
O2—Mo1—S2	105.96 (4)	C9—O3—Mo1	124.0 (2)
S1—Mo1—O3	80.68 (5)	C7—O3—Mo1	125.8 (2)
S2—Mo1—O3	74.70 (5)	C9—O3—Mo1^i^	115.1 (2)
S2—Mo1—S1	71.612 (11)	C7—O3—Mo1^i^	119.6 (2)
O2—Mo1—Mo1^i^	107.62 (3)	Mo1—O3—Mo1^i^	54.01 (4)
O1^i^—Mo1—Mo1^i^	49.26 (3)	C7—C6—C8	104.6 (3)
O1—Mo1—Mo1^i^	48.87 (3)	C7—C6—H6X	110.8
S2—Mo1—Mo1^i^	130.072 (11)	C8—C6—H6X	110.8
S1—Mo1—Mo1^i^	132.947 (10)	C7—C6—H6Y	110.8
Mo1^i^—Mo1—O3	70.02 (5)	C8—C6—H6Y	110.8
C1—S1—Mo1	87.29 (4)	H6X—C6—H6Y	108.9
C1—S2—Mo1	87.53 (4)	O3—C7—C6	105.9 (3)
Mo1^i^—O1—Mo1	81.87 (4)	C6—C7—Mo1	141.2 (2)
C1—N1—C4	122.27 (11)	O3—C7—H7X	110.6
C1—N1—C2	121.66 (11)	C6—C7—H7X	110.6
C4—N1—C2	116.06 (11)	Mo1—C7—H7X	92.6
N1—C1—S1	123.18 (10)	O3—C7—H7Y	110.6
N1—C1—S2	123.24 (10)	C6—C7—H7Y	110.6
S1—C1—S2	113.54 (7)	Mo1—C7—H7Y	89.4
N1—C1—Mo1	176.95 (11)	H7X—C7—H7Y	108.7
S1—C1—Mo1	56.96 (4)	C9—C8—C6	102.8 (3)
S2—C1—Mo1	56.60 (4)	C9—C8—H8X	111.2
N1—C2—C3	110.82 (11)	C6—C8—H8X	111.2
N1—C2—H2A	109.5	C9—C8—H8Y	111.2
C3—C2—H2A	109.5	C6—C8—H8Y	111.2
N1—C2—H2B	109.5	H8X—C8—H8Y	109.1
C3—C2—H2B	109.5	O3—C9—C8	104.7 (3)
H2A—C2—H2B	108.1	C8—C9—Mo1	138.4 (3)
C2—C3—H3A	109.5	O3—C9—H9X	110.8
C2—C3—H3B	109.5	C8—C9—H9X	110.8
H3A—C3—H3B	109.5	Mo1—C9—H9X	82.1
C2—C3—H3C	109.5	O3—C9—H9Y	110.8
H3A—C3—H3C	109.5	C8—C9—H9Y	110.8
H3B—C3—H3C	109.5	Mo1—C9—H9Y	100.8
N1—C4—C5	111.21 (12)	H9X—C9—H9Y	108.9
N1—C4—H4A	109.4		
			
O2—Mo1—S1—C1	103.85 (6)	O1^i^—Mo1—O3—C7	50.7 (3)
O1^i^—Mo1—S1—C1	−147.26 (6)	O1—Mo1—O3—C7	155.4 (3)
O1—Mo1—S1—C1	−49.51 (7)	S2—Mo1—O3—C7	−112.8 (3)
S2—Mo1—S1—C1	0.95 (5)	S1—Mo1—O3—C7	−39.4 (3)
Mo1^i^—Mo1—S1—C1	−127.18 (5)	Mo1^i^—Mo1—O3—C7	103.2 (3)
O3—Mo1—S1—C1	−75.92 (7)	O1^i^—Mo1—O3—Mo1^i^	−52.44 (3)
O2—Mo1—S2—C1	−99.06 (6)	O1—Mo1—O3—Mo1^i^	52.22 (3)
O1^i^—Mo1—S2—C1	58.14 (7)	S2—Mo1—O3—Mo1^i^	144.06 (3)
O1—Mo1—S2—C1	154.38 (6)	S1—Mo1—O3—Mo1^i^	−142.59 (3)
S1—Mo1—S2—C1	−0.94 (5)	C9—O3—C7—C6	21.9 (3)
Mo1^i^—Mo1—S2—C1	130.25 (5)	Mo1—O3—C7—C6	−176.6 (2)
O3—Mo1—S2—C1	84.12 (7)	Mo1^i^—O3—C7—C6	−111.7 (3)
O2—Mo1—O1—Mo1^i^	98.92 (4)	C9—O3—C7—Mo1	−161.5 (3)
O1^i^—Mo1—O1—Mo1^i^	−13.32 (5)	Mo1^i^—O3—C7—Mo1	64.91 (18)
S2—Mo1—O1—Mo1^i^	−155.46 (3)	C8—C6—C7—O3	1.4 (4)
S1—Mo1—O1—Mo1^i^	−108.31 (4)	C8—C6—C7—Mo1	−1.7 (6)
O3—Mo1—O1—Mo1^i^	−80.47 (6)	O2—Mo1—C7—O3	−173.53 (19)
O1^i^—Mo1—O2—Mo1^i^	52.06 (3)	O1^i^—Mo1—C7—O3	−122.3 (3)
O1—Mo1—O2—Mo1^i^	−51.33 (3)	O1—Mo1—C7—O3	−23.1 (3)
S2—Mo1—O2—Mo1^i^	−142.50 (2)	S2—Mo1—C7—O3	63.8 (3)
S1—Mo1—O2—Mo1^i^	143.34 (2)	S1—Mo1—C7—O3	136.9 (3)
C4—N1—C1—S1	−172.85 (11)	Mo1^i^—Mo1—C7—O3	−71.1 (3)
C2—N1—C1—S1	7.5 (2)	O2—Mo1—C7—C6	−168.3 (3)
C4—N1—C1—S2	4.7 (2)	O1^i^—Mo1—C7—C6	−117.0 (5)
C2—N1—C1—S2	−174.93 (10)	O1—Mo1—C7—C6	−17.8 (4)
Mo1—S1—C1—N1	176.36 (12)	S2—Mo1—C7—C6	69.0 (4)
Mo1—S1—C1—S2	−1.40 (7)	S1—Mo1—C7—C6	142.1 (5)
Mo1—S2—C1—N1	−176.35 (12)	Mo1^i^—Mo1—C7—C6	−65.9 (4)
Mo1—S2—C1—S1	1.40 (7)	O3—Mo1—C7—C6	5.2 (3)
O2—Mo1—C1—S1	−86.15 (6)	C7—C6—C8—C9	−23.1 (5)
O1^i^—Mo1—C1—S1	37.22 (6)	C7—O3—C9—C8	−37.5 (4)
O1—Mo1—C1—S1	152.10 (4)	Mo1—O3—C9—C8	160.5 (2)
S2—Mo1—C1—S1	−178.47 (8)	Mo1^i^—O3—C9—C8	98.3 (3)
Mo1^i^—Mo1—C1—S1	93.83 (5)	C7—O3—C9—Mo1	161.9 (3)
O3—Mo1—C1—S1	95.97 (7)	Mo1^i^—O3—C9—Mo1	−62.18 (15)
O2—Mo1—C1—S2	92.31 (6)	C6—C8—C9—O3	36.8 (4)
O1^i^—Mo1—C1—S2	−144.31 (4)	C6—C8—C9—Mo1	54.3 (6)
O1—Mo1—C1—S2	−29.43 (6)	O2—Mo1—C9—O3	172.71 (17)
S1—Mo1—C1—S2	178.47 (8)	O1^i^—Mo1—C9—O3	27.7 (3)
Mo1^i^—Mo1—C1—S2	−87.70 (6)	O1—Mo1—C9—O3	126.7 (3)
O3—Mo1—C1—S2	−85.56 (7)	S2—Mo1—C9—O3	−128.3 (3)
C1—N1—C2—C3	90.63 (16)	S1—Mo1—C9—O3	−59.5 (3)
C4—N1—C2—C3	−89.02 (15)	Mo1^i^—Mo1—C9—O3	75.6 (3)
C1—N1—C4—C5	98.86 (16)	O2—Mo1—C9—C8	143.6 (4)
C2—N1—C4—C5	−81.49 (16)	O1^i^—Mo1—C9—C8	−1.4 (4)
O1^i^—Mo1—O3—C9	−150.6 (3)	O1—Mo1—C9—C8	97.6 (5)
O1—Mo1—O3—C9	−46.0 (3)	S2—Mo1—C9—C8	−157.4 (5)
S2—Mo1—O3—C9	45.9 (3)	S1—Mo1—C9—C8	−88.6 (5)
S1—Mo1—O3—C9	119.2 (3)	Mo1^i^—Mo1—C9—C8	46.5 (4)
Mo1^i^—Mo1—O3—C9	−98.2 (3)	O3—Mo1—C9—C8	−29.1 (4)

Symmetry codes: (i) −*x*, *y*, −*z*+1/2.

### Hydrogen-bond geometry (Å, °)

**Table:**

*D*—H···*A*	*D*—H	H···*A*	*D*···*A*	*D*—H···*A*
C2—H2A···O2^ii^	0.99	2.57	3.2687 (17)	127
C4—H4B···O2^iii^	0.99	2.49	3.2443 (17)	133

Symmetry codes: (ii) −*x*+1/2, *y*+1/2, −*z*+1/2; (iii) −*x*+1/2, −*y*+1/2, −*z*.
